# Arboreal camera trap reveals the frequent occurrence of a frugivore-carnivore in neotropical nutmeg trees

**DOI:** 10.1038/s41598-022-11568-z

**Published:** 2022-05-07

**Authors:** Marie Séguigne, Opale Coutant, Benoît Bouton, Lionel Picart, Éric Guilbert, Pierre-Michel Forget

**Affiliations:** 1 Département Adaptations du Vivant, Muséum National d’Histoire Naturelle, Mécanismes Adaptatifs et Evolution MECADEV – UMR 7179, CNRS-MNHN, Labex DRIIHM, 1 avenue du petit château, 91800 Brunoy, France; 2 Laboratoire d’Écologie Fonctionnelle et Environnement - UMR 5245, Université Toulouse III Paul Sabatier, 31062 Toulouse cedex 9, France; 3 Laboratoire Évolution et Diversité Biologique - UMR 5174, Université Toulouse III Paul Sabatier, 31062 Toulouse cedex 9, France; 4 Hévéa, 78310 Maurepas, France; 5 A l’Ecoute de l’Arbre, 60650 Lachapelle-aux-Pots, France

**Keywords:** Biodiversity, Conservation biology, Tropical ecology

## Abstract

Arboreal and flying frugivorous animals represent primary dispersers in the Neotropics. Studies suggest a possible compensation for the loss of large species by smaller ones with expanding rampant anthropogenic pressures and declining populations of larger frugivores. However, studies on seed dispersal by frugivores vertebrates generally focus on the diurnal, terrestrial, canopy, and flying species, with the nocturnal canopy ones being less studied. Setting camera traps high in the canopy of fruiting nutmeg trees revealed for the first time the high frequency of the kinkajou (*Potos flavus*, Schreber, 1774, Procyonidae), an overlooked nocturnal frugivore species (Order Carnivora) in the Guianas. The diversity of the fruit species consumed by the kinkajou calls for considering it as an important seed disperser. The overlap of the size of seeds dispersed by frugivores observed in nutmeg trees suggests that the small (2–5 kg) kinkajou may compensate for the loss of large (5–10 kg) frugivorous vertebrates in the canopy. Camera traps visualise how the kinkajou is adapted to forage in the nutmeg tree crown and grab the fruit. Such information is vital for conservation because compensation of seed dispersal by small frugivores is crucial in increasing anthropogenic stressors.

## Introduction

Frugivore vertebrates eat fruit and disperse plant diaspores, defecating, regurgitating, or spitting out seeds beyond the vicinity of the parent tree, where it is less advantageous to develop as a seedling (Janzen-Connell model), given the higher risks of competition and mortality by pathogens and other predators^1–3^. Therefore, seed dispersal by fruit-eaters plays a crucial role in shaping the seed-seedling shadow, animal-mediated seed dispersal being a primary driving mechanism of tropical forest dynamics and maintaining diversity in tropical forests^4–7^.

Studies on seed dispersal by vertebrates generally focus on the diurnal, terrestrial, canopy, and flying frugivore species that are easy to observe from the ground using binoculars. For instance, in neotropical forests, large body-sized primates and birds represent the primary groups responsible for dispersing small-to-large seeds^8–15^. However, hunters also prefer these animals, sold for meat, medicine, game trophies, and pets^16,17^. Studies often focus on bats regarding nocturnal frugivore species, highlighting their crucial function for small-seeded plant dispersal during the early stages of succession^18,19^. Other nocturnal canopy frugivores are less studied, even though, being a diverse assemblage of vertebrates, they may play an important role in the dispersal for many plants according to their food preferences^20^. For example, a recent study on nocturnal neotropical oilbirds (*Steatornis caripensis*, von Humbold 1817) showed their ability to disperse large seeds (29 mm in width) up to 10 km on average, suggesting that they may perform similar ecological roles to some of the extinct megafauna, despite the oilbirds’ smaller size^21^. Nocturnal frugivores have also received some further attention. They are acknowledged as seed dispersers in various tropical rainforests, such as the Viverrid palm civets (*Paradoxurus hermaphroditus*, Pallas 1977) and binturong (*Arctictis binturong*, Raffles 1821) in Asia^22,23^, and the Procyonid kinkajou (*Potos flavus*, Shrebber 1774) in the neotropics^20,24,25^. Although more research considers nocturnal frugivores, there is still a gap in knowledge regarding their role in seed dispersal because of the difficulty of studying them. It can be highlighted by the recent discovery of a new olingo species (*Bassaricyon* spp.)^26^, another arboreal Procyonid in Ecuador, and the potential existence of different species within the genus *Potos*^27^. However, the development of safer single-rope tree climbing^28^ and remote camera trapping^29^ offers new opportunities to observe and analyse their behavioural ecology in treetops. Indeed, Gregory et al*.* (2017) recorded that 94% of canopy activity in natural branches happened at night^30^.

Nutmeg tree species (Myristicaceae) form a pantropical family that heavily relies on animals for their seed dispersal, thus are excellent ecological models for the study of animal-plant interactions^31^. In neotropical biomes, primates and toucans are the primary consumers and dispersers of nutmeg tree seeds^8,9,11,12,32–34^. Facing an increase in anthropogenic pressure^27^, populations of large arboreal frugivores in tropical forests are threatened^35–37^, leading to the downsizing of frugivore communities^38,39^. Hence, it is expected the seed dispersal process to be altered, resulting in a decline in animal-dispersed tree species diversity^40,41^. Nevertheless, studies suggest the existence of possible compensation for the disappearance of large mammalian frugivores with replacement by smaller ones, among them kinkajous in disturbed forest habitats^8,42,43^. Although kinkajous rarely forage in fruiting nutmeg trees in a study in Central America^34,44^, they are known to consume and disperse nutmeg tree seeds in the Amazonian forests of the Guianas^45,46^. Observations in neotropical nutmeg (*Virola* spp*.*) tree species^45^ showed that seed-dispersal for nutmeg trees is not disrupted when large diurnal, arboreal frugivores are missing^8^. Given that kinkajou has a body mass (2–5 kg) similar to that of the diurnal frugivores brown capuchin (*Sapajus apella*, Linnaeus 1758)^20^ and a dietary diversity overlapping that of the black spider monkey (*Ateles paniscus*, Linnaeus 1758), we assume that kinkajous are significant seed dispersers ^24^. Here, we hypothesised that kinkajous might compensate for the loss of other larger arboreal frugivores among plant species that share a diversified array of consumers.

**Figure 1:**
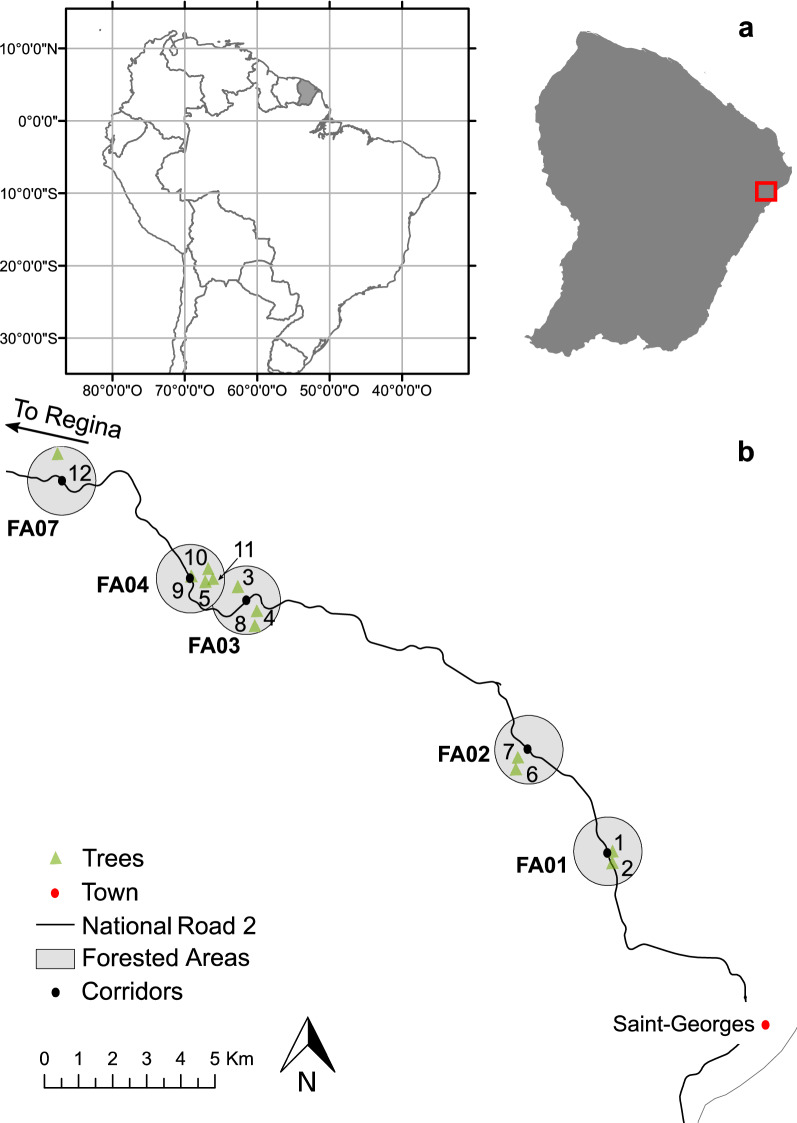
Study trees in the forested areas (FA) surrounding the ecological corridors along the National 2 Road between Régina and Saint-Georges in French Guiana (see further description in Coutant et al*.* 2022^57^). The map was edited on ArcMap software version 10.6.1 (https://www.esri.com/).

To test this hypothesis, we used camera traps, which are an effective method for studying arboreal fauna^29,47–49^. The cost of detecting nocturnal animals such as kinkajous is reduced by half when using camera traps instead of line transect survey^50^. In previous studies, frugivores were observed in nutmeg trees from the ground^12^. Others followed animals using arboreal camera traps studies in trees using easy and safe climbing gear ^29,47,50^. We, therefore, set up camera traps directly in two well-known *Virola* species (height between 30 and 40 m) in the forest near the newly-constructed National 2 Road (Fig. 1). We sampled 12 trees during the fruiting peak season (December-January): *Virola kwatae*, Sabatier 1997 (7 trees) and *V. michelii*, Heckel 1898 (5 trees). Through analysis of camera trap images, this study aimed to assess the occurrence of the frugivore kinkajou in *Virola* trees compared to other diurnal and nocturnal counterparts. Because the varying capacities of foraging animals to disperse seeds creates selective pressures on seed size^51^, we supplement our results with a literature-review-based analysis of the average seed size dispersed by small- to medium- and large-bodied frugivores.

## Results

**Figure 2:**
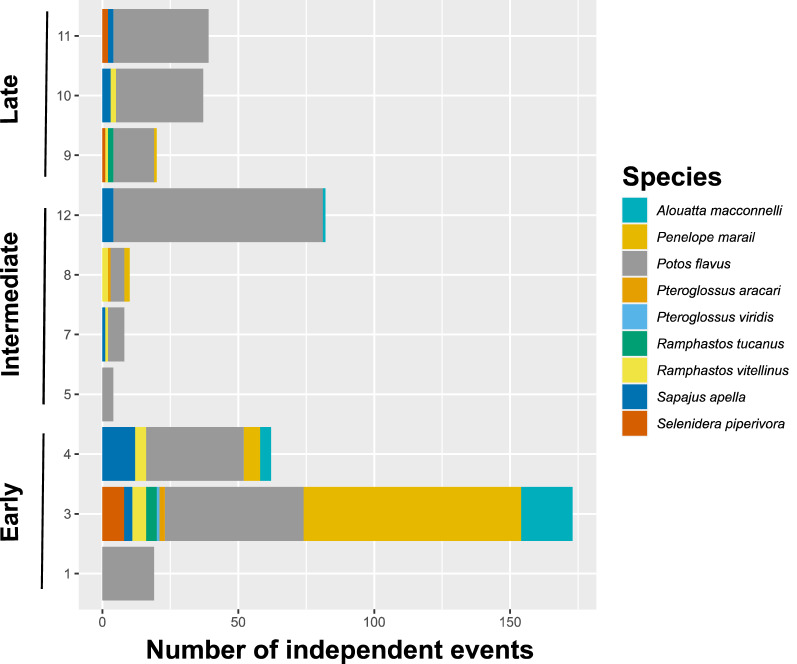
Number of independent events by tree for the nine main frugivores recorded as visiting the canopy of nutmeg trees between 12/01/19 and 01/24/20 in National 2 Road in French Guiana. Trees were grouped by their fruiting statues, either “early”, “intermediate”, or “late”. Trees 2 and 6 are not represented because no frugivores were recorded in their crown.

The camera trap survey was for 1320 trap nights (55 days × 12 trees × 2 camera traps), and we observed 24 identified species and unidentified species grouped per order visiting *Virola* trees (Fig. S1). Fruiting was staggered, with early and late-fruiting individual trees (Tab. S1). The analysis of 162,885 images on 11 fruiting trees and one flowering tree produced 587 independent events with high variability between trees (Fig. S1, S2, Tab. S1). Photographs 30 min apart were considered independent events, as usually defined in literature^51–54^. We recorded nine frugivorous species known to be regular *Virola* seed dispersers (Fig. 2, Tab. S2, Fig. S3). On the one hand, all known frugivores visited the single early-fruiting *V. michellii* tree (tree #3), which had 29% (N = 171) of all independent events. Contrastingly, no frugivores visited the late-fruiting *V. michellii* (tree #2) and the non-fruiting *V. kwatae* tree (tree #6) (Fig. 2, Tab. S2). The kinkajou was the most frequent fruit-eating species overall, representing 48% (N = 280) of the events. The guan (*Penelope marail*, Statius Muller 1776) ranked second (15%, N = 89 independent events), including 80 events in the same tree (tree #3, Fig. 2, Tab. S2). Howler monkey (*Alouatta macconnelli*, Linnaeus 1766) and brown capuchin (*Sapajus apella)* each represented 4% of events, while the five species of toucans combined accounted for 6% of events, the channel-billed toucan (*Ramphastos vitellinus*, M.H.K. Lichtenstein 1823) being the primary (50%) species in nutmeg trees.

**Figure 3:**
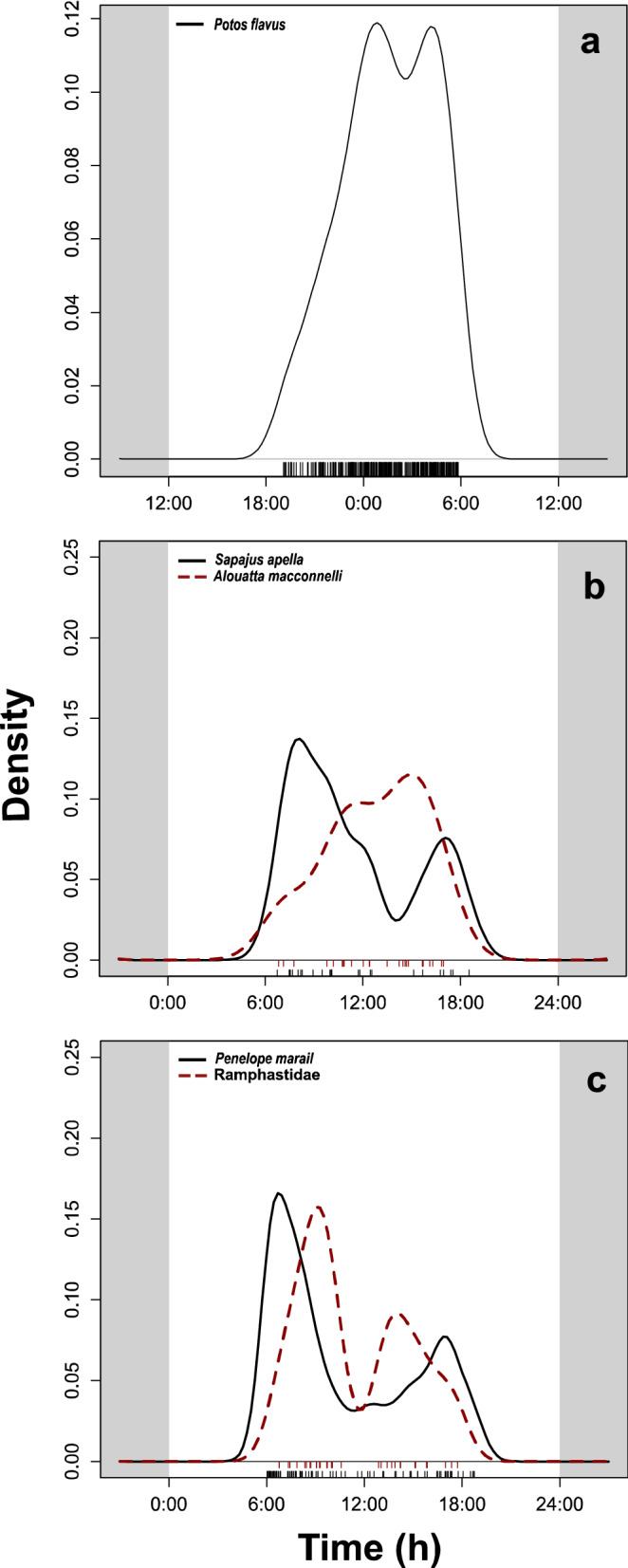
Night activity of (**a**) kinkajou (*Potos flavus*), and daily activity of (**b**) primates (*Alouatta macconnelli* and *Sapajus apella*) and (**c**) birds (*Penelope marail* and Ramphastidae, toucans) in *Virola* spp trees near National 2 Road between 12/01/19 and 01/24/20. Small bars (x-axis) represent recorded events: the greater the number of events observed at a given time, the greater the density (y-axis).

Kinkajou activity peaked between 00:00 and 05:30 (Fig. 3a). Diurnal frugivores showed two activity peaks during the day, except for *A. macconnelli,* primarily active around 15:00 (Fig. 3b). *Sapajus apella* had a maximum activity around 08:00 and 17:30 (Fig. 3b). *Penelope marail* and toucans showed a similar pattern of activity, but *P. marail* was predominantly active at dawn and dusk, whereas toucans were active around 09:00 and 14:00 (Fig. 3c).

**Figure 4:**
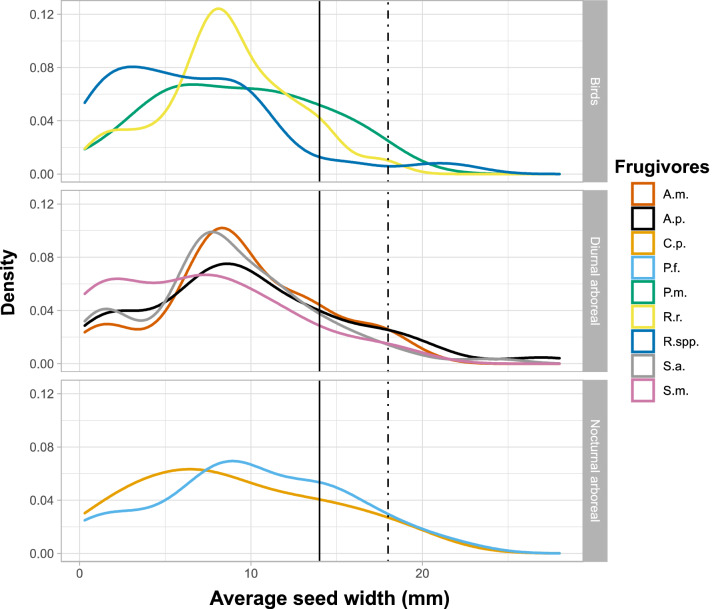
Density plot of average seed width (mm) dispersed by major neotropical arboreal frugivores. Data are obtained from literature based on studies in French Guiana, except for data on Ramphastidae spp., which were compiled from studies in the Atlantic Forest. Solid black line = average *Virola michelii* seed width. Dashed line = average *Virola kwatae* seed width. *A.m.* = *Alouatta macconnelli*; *A.p.* = *Ateles paniscus; C.p.* = *Caluromys philander; P.f.* = *Potos flavus; P.m.* = *Penelope marail; R.r.* = *Rupicola rupicola;* R. spp. = Ramphastidae spp.*; S.a.* = *Sapajus apella; S.m.* = *Saguinus midas*. Data for *Virola* seed size are from Ratiarison & Forget (2013)^12^.

Based on estimates from the literature, the overall distribution of average seed width dispersed by the frugivores that visit *Virola* trees shows similar patterns among species and groups (Fig. 4). Dunn tests corroborate these observations and highlight trends among toucans to disperse seeds significantly smaller (median width and length = 5.77 × 8.5 mm) than other larger vertebrate species (details in Tab. S3, S4, S5, S6, S7, and Fig. S4). The arboreal vertebrate species show similar patterns in the distribution of dispersed seed dimensions (Fig. 4, S4) with similar median seed width (*A. paniscus* = 9 mm; *P. flavus* = 10 mm; *A. macconnelli* = 9 mm; *S. apella* = 8 mm) and similar median seed length (*A. paniscus* = 15 mm; *P. flavus* = 15.5 mm; *A. macconnelli* = 15.5 mm; *S. apella* = 14.5 mm).

## Discussion

Kinkajou (*Potos flavus*) frequently visited the two species of *Virola* nutmeg trees during the peak fruiting season at the study site (see Tab. S2, Fig. S5). Our study shows for the first time that kinkajous are very common in nutmeg trees. At the same time, the primary consumer (i.e., black spider monkey, *Ateles paniscus*) is nearly absent in the study forest.

The analysis of the activity of kinkajous in trees suggests that fruiting nutmeg trees are often visited when fewer alternative fruit resources are available in the early rainy season, a period with low fruit production^55,56^. The observed occurrence of kinkajous in fruiting *Virola* trees maybe related to a deficit of the larger diurnal, arboreal consumer, i.e. the spider monkey, at the study area. In other forests, such as the protected Nouragues Nature Reserve^25^, spider monkeys are the most common diurnal visitors of fruiting *Virola* trees between October and March^12^. When spider monkeys are scarce, there is thus a greater abundance of ripe, dehiscent fruit that may reach maturity and dehisce in the canopy during daily hours, exposing red aril^43^. The consumed part is then easily accessible to other diurnal and nocturnal consumers, i.e. birds and carnivores, that would otherwise be unable to open fruit when spider monkeys are present. Confirmation that kinkajou visitation rates are directly impacted by the presence of spider monkeys requires a camera trap study where both species are present (ongoing studies).

Consequently, fruit surveys on the ground had shown a more significant proportion of *Virola* fruit with single valve husks at disturbed vs control forests^57^. However, the difference in seed removal rates across sites suggested that secondary consumers played an important role as alternative seed dispersers (e.g., toucans), eventually compensating for the disappearance of the primary seed dispersers^57^. This study shows that the nocturnal frugivore kinkajou complements the effects of birds as seed dispersers of *Virola* seeds. During the first hours after sunset, kinkajou travel from its diurnal shelter to feed on *Virola* fruit (Fig. 3a) and return before dawn to their daily sleeping roost located further away from the feeding trees^46^. This behaviour has never been documented since studies of frugivory traditionally focus on diurnal frugivores.

The review of the size of seeds dispersed by the main frugivores observed and known to forage in nutmeg trees showed the significant overlap between frugivores. This result supports the hypothesis that smaller animal species might compensate for the absence of large ones. In particular, the pattern of seed size highlights the role of kinkajous, howler monkeys, and brown capuchins as potential replacement species for spider monkeys in human-affected forest areas. Moreover, studies have shown that kinkajous disperse seeds on average 200 m away and up to 300 m from parent trees^46^. In comparison, howler monkeys and brown capuchins disperse seeds up to 390 m and 225 m, respectively on average^58^, within more extensive home ranges than kinkajous^59,60^. On the other hand, spider monkeys seem to disperse seeds between 150 and 250 m, depending on sites^58^.

Although these observations support the possible compensatory role of these three species, in the absence of spider monkeys for seed dispersal, howler monkeys and brown capuchins remain preferred hunting targets^36^. In addition, howler monkeys and brown capuchins also only shared 35 to 59% overlap in fruit feeding species with spider monkeys^61^ and presented a more diversified diet composed of leaves and arthropods^59,60^ at that time, making them less dependent on the availability of ripe fruits. Contrastingly, kinkajous are barely hunted and remain abundant in many forest areas^62^. As a frugivore generalist and opportunist, kinkajous can eat similar plants as black spider monkeys with a similar digestive retention time^63^ in the Guianas, as observed for the Central American spider monkey in Panama^24^. Like neotropical primates, kinkajous are adapted to forage into the canopy using their prehensile tail^64^. It allows them to move on small branches^65^ where they can reach and grasp fruit using their forepaws, teeth, snout or nose^66,67^. All previous facts support the hypothesis that kinkajous may play a significant role in compensating seed dispersal of nutmeg trees and, more generally, for a fraction of the fruits usually consumed by spider monkeys.

These observations highlight the importance of studying the role of kinkajous and other nocturnal species such as olingos (*Brassaricyon gabbii*, Allen 1876) in seed dispersal. Olingos are understudied as they were traditionally confused with kinkajous because of their similar appearance. However, a study in Panama suggested that olingos share some ecological and behavioural traits with kinkajous and may disperse ingested seeds^68^. Since almost no studies have focused on their potential role for seed dispersal, there is thus a clear need to increase research on this group to better understand their ecological importance, especially in disturbed forests. Similarly, little is known about other diurnal frugivorous carnivores such as the coati (*Nasua nasua*, Linnaeus 1766), which could also play a key role in seed dispersal in fragmented forests where it can persist^69^, as well as the tayra (*Eira barbara*, Linnaeus 1758) that could also be involved in supporting seed dispersal processes as it also consumes fruits^70^.

In this study, another arboreal, nocturnal frugivore was recorded once: *Caluromys philander* (Linnaeus 1758). This marsupial consumes 25 different fruit species in the forests of Cabassou, French Guiana, including three nutmegs species^20^. At the Piste de Saint-Élie, French-Guiana, *C. philander* was not eating any *Virola* fruits. Still, it seems to share a large part of its diet composed of flowers and small-seeded fruits during the dry and wet season, respectively, with kinkajous^45^. In addition, our seed-size review demonstrated its capability to disperse seeds slightly smaller than those of kinkajous (not significant, Tab. S4,S6, S7). Even though *C. philander* was only observed once here, its presence in a wide range of habitats in French-Guiana^71^ without being threatened by hunting suggests that it could play an important role in dispersing small seeds^72^, as indicated for small rodents^73^.

Seed size can be a fundamental threshold level for seed dispersal, especially for birds. This review highlighted a significant trend for toucans and the Guianan Cock-of-the-rock (*Rupicola rupicola*, Linnaeus 1766) to consume and disperse smaller seeds. Although Cock-of-the-rock was not observed in the study forest, this smaller bird is known to disperse large seeds (Tab. S7), including species consumed by spider monkeys, toucans, and kinkajous (P-M. Forget, pers. obs). Larger birds swallow these seeds, such as the guan (*Penelope marail*). The gizzard of this species is only slightly muscular, allowing the seed to pass through the whole digestive tract without being crushed^74,75^. Conclusion on toucans need to be tempered and could be explained by a possible site effect. Indeed, Mata Atlantica data were used to describe seed size consumed by toucans. However, seeds from Mata Atlantica are possibly smaller than in French Guiana, which could bias our results. This highlights the clear need for studies to evaluate their role in seed dispersal compensation.

This study points out the importance of smaller vertebrates for seed dispersal and explores a new approach for studying arboreal frugivores with camera traps. Beyond the difficulty of finding the most suitable location for setting up cameras to maximise the chance of capturing animals, implementing this methodology involves very tedious work that requires time, climbing skills, specific equipment to access the upper branches in the tree crown, and the presence of at least two people capable of climbing to ensure safety^28^. During this study, approximately two trees were equipped/unequipped at a height over 30 m per day by two climbers. The number of climbs is a limitation that complicates the ability to increase the number of fruit trees and sites covering a large area, i.e., that of the home range of frugivores to be inventoried far from forest access points. In addition, even when fixed directly to the trunk or the main branches, unavoidable swinging movements of the crown can cause many triggers of non-target stimuli^49^, especially during the day when the wind blows. Saturation of the memory card follows, plus battery draining faster than expected, making it impossible to maintain traps in place for more than two months without a checkup. It also underestimates frugivorous bird frequency that perches on smaller branches away from the trunk. For instance, toucans were recorded only with 33 independent events while they are known to be a regular consumer of *Virola* fruit and little affected by hunting in this area^8,9,43^. Future studies should consider setting up supplementary cameras in adjacent trees to observe frugivorous birds as Zhu et al*.* (2021)^76^ did and succeeded in recording many of their interactions with fruits.

Despite these constraints, arboreal camera traps allowed for continuous, non-invasive observation of animals in the canopy, both day and night, providing a wealth of information (diversity, activities, and behaviour) about the frugivore guild of *Virola* spp. in the area. Specifically, this method effectively studied nocturnal frugivores, such as kinkajous, in *Virola* spp. and many other staple fruit resources for arboreal mammals and birds (e.g., *Tetragastris* spp.^13,44^). The effectiveness of this method is consistent with the finding of a study from Bowler et al*.* (2017)^47^ who were also able to obtain a large number of images of kinkajous without ever observing it during counts and transect observations. Schipper et al. (2007)^77^ showed that standard flash photography in camera traps leads to avoidance behaviour in kinkajou. Here, the use of red infrared moving sensor camera traps suggested by Schipper et al*.* (2007)^77^ minimised disruption to the foraging behaviour of the animals, as evidenced by a large number of kinkajous captured. It is also the case for primates who look at the lens, sometimes touching it (*S. apella*), but do not linger on it and do not seem bothered by camera traps when feeding. Therefore, it favours using these devices to characterise arboreal fauna. This method is currently promising for studies in the canopy^29,40,47–49,51^.

During this study, thanks to many recorded events for kinkajous (N = 280, during 1320 trap nights), we showed how arboreal camera trapping protocols in targeted fruiting trees could complement the classical radio-tracking of animals foraging in their home range. Firstly, camera traps can help survey several independent populations of frugivores in various habitats and feeding trees. In contrast, radio-tracking is often restricted to fewer individuals in their respective home ranges. Secondly, one can further document interactions with other frugivores species in the canopy within-tree and across plant species during their daily or nocturnal activities. And finally, camera traps record all behavioural attitudes of animals, whether they are moving along the trunk or within the branches of the canopy, handling and consuming fruit, swallowing seeds, resting, or simply visiting the treetops. These observations are simply impossible from the ground at night.

Cameras might, therefore, successfully be implemented in addition to line transects^47^, eco-acoustics^9^, or radio-tracking^10,42^ to obtain the best overview of the diversity and behavioural ecology of the arboreal and flying fauna. Camera traps revealed the high frequency of an overlooked nocturnal species for the first time, suggesting its potential role in compensating the loss of other large frugivorous vertebrates such as black spider monkeys for nutmeg trees seed dispersal. Camera traps also allowed visualising how frugivore species foraging within the canopy reach ripe fruit and how they behave, highlighting the advantage of primates, which can pick, open, and peel fruit. Kinkajous handle the dehiscent fruit, and swallow seed and aril. They may pull the twig with the open fruit, and swallow the arilled seed. Then, they later defecate cleaned seeds in their droppings, similarly to primates such as spider monkeys, capuchins and howler monkeys (Forget, P-M, pers. obs.; dual observation along with Didier Julien-Laferrière in February 1986 at Piste de Saint-Elie). Such information is vital for conservation because compensation for seed dispersal by small frugivores is crucial in the context of increasing anthropogenic disturbance.

To conclude, the extension of protocols such as this one to other fruiting trees is essential to measure the impact of human disturbance on the ecology and dynamics of tropical forests. Fruit-eating species compensation seems to be mainly dependent on the diet of the smallest frugivores, as pointed out by Boissier et al*.* (2020)^8^, who observed a significant impact of anthropisation on the dispersal of Sapotaceae. In contrast, it was more moderate for trees of the families Myristicaceae and Burserasae, whose fruits are mainly consumed by mammals and birds^13,78^. It is also expected to see fruiting plants with the largest seeds most affected by the loss of larger frugivores, as just a few large frugivores (*A. paniscus*) can consume and disperse these seeds, whereas other species such as kinkajous are too small to consume these seeds (see Fig. 4, S4). The use of camera traps in anthropised areas appears necessary to characterise the canopy fauna more completely by including all nocturnal species likely to participate in seed dispersal processes. Identifying these species is a crucial point that would allow a better understanding of the sharing of resources between diurnal and nocturnal species and a better understanding of seed dispersal compensation processes made possible by the functional redundancy of the ecosystem.

## Material and methods

### Study site

The study was conducted in French Guiana (Fig. 1). The climate is equatorial and characterised by turns between a wet season from December to July and a dry season from August to November. Peak fruiting period occurs between March and May^55,56^. The study site was based in an anthropised forest in Eastern French Guiana (Fig. 1). More precisely, sampled trees were located near (< 1 km) the ecological corridors implemented along the National 2 Road (RN2 in french) that facilitate animal passage on the ground or in the canopy (Fig. 1). Because the road follows the hilly topography of the forest, these corridors are at elevations ranging from 30 to 90 m.

### Study tree species

*Virola kwatae* and *V. michelii* (Myristicaceae) are two dioecious trees of the canopy in the Guiana Shield with an associated frugivores community well studied in the Nouragues Nature Reserve^12,79,80^ and a disturbed forest^8,43^. *Virola kwatae* is more likely to be found on hills and forests on steep slopes^80^, while *V. michelii* is more common and can be found in high densities in secondary areas^20^. Both species fruit synchronously between November and March before the forest fruiting peak between March and May^55,79,80^. Fruiting duration is ca. two months and 3–4 months in *Virola michelii* and *V. kwatae*, respectively^78,79^. They produce a dehiscent capsule-like fruit with two valves protecting a seed covered by a red lipid-rich aril exposed when the fruit reaches maturity^12,79,80^. Seeds of *V. kwatae* are larger (seed size average: 2.8 × 1.8 cm) than seeds of *V. michelii* (seed size average: 2.0 × 1.4 cm). In this study, 12 trees were sampled in five forest areas (FA) located 1000 m from the road and corridors (Fig. 1b). The first forest area (FA01) is the closest to Saint-Georges with the lowest elevation (30–50 m), and the last forest area (FA07) is the farthest and is slightly higher (70–90 m).

### Camera trapping protocol

Three models of Reconyx® camera traps were used: HC600 Hyperfire, XR6 Ultrafire, and Hyperfire2. The red infrared moving sensor and the absence of a white flash during shooting do not disturb the behaviour of nocturnal animals^77^. We set the HC600 and Hyperfire2 traps to take five photographs upon movement detection, while the XR6 Ultrafire was programmed to take three pictures and one video. We activated the video function for experimental purposes to supplement the information gathered from the photographs by documenting the behaviour of the foraging animals. We placed 34 camera traps (including 21 HC600 Hyperfire, 8 Hyperfire2, and 5 XR6 Ultrafire) in the crowns of 11 fruiting trees (6 *Virola kwatae* and 5 *V. michelii* trees). In addition, cameras were set in one non-fruiting tree (*V. kwatae*) to test for a possible attractive effect of the fruits on the other fertile trees. Except for two trees with two camera traps, all others had three traps in their canopy on the trunk and main branches, all between 30 and 40 m high. We carried out a camera trap survey between 11/25/19 and 12/01/19, while removal took place between 1/24/20 and 1/28/20. Upon removal of the traps, the fruiting status of the trees was characterised and categorised as follows:

- A: early fruiting, many fruits on the ground and few fruits in the canopy;

- B: intermediate fruiting, many fruits on the ground and in the canopy;

- C: late fruiting, few fruits on the ground, and most fruits in the canopy;

- D: no fruiting.

### Camera trapping analysis

Following the protocol of Niedballa et al*.* (2016)^81^, data (images) were first extracted and organised using the camtrapR® package on Rstudio® Version 1.1.463. Digikam® software tagged images displaying an animal to add the identified species name to the image's metadata. In some cases, the individuals photographed did not allow for species identification, so we tagged the pictures with the common name of the animal observed (e.g., sloth, large marsupial, large rodent). Photographs that did not identify a particular animal (blurred image, only part of the organism photographed) were classified as unidentified species. Each identification was then used to determine the independent events obtained. An event is defined by recording an individual by a camera trap. It is considered independent of another at least 30 min apart, as is usually expressed in literature^51–54^. To avoid pseudo-replicates, counting events was performed by considering traps in the same tree as a single trap. If an individual of a species is recorded by two camera traps of the same tree within a 30-min interval, then only one event is counted. Only one event is counted if more than one individual is observed in the image. Because not all trees had the same number of camera traps, we analysed only the pictures collected by two traps on each tree to ensure consistent sampling effort among the 12 sites. The two traps that provided the most images were chosen to select them. To standardise the period of data recording on each tree, only images obtained between 01/12/19 starting at 18:00 and 24/01/20 until 06:00 were analysed, a period of 55 days. The overlap® package^82^ was used to produce activity plots of the main frugivores. Graphs were centred either at noon or midnight depending on their activity (e.g., kinkajou is a nocturnal frugivore, so its figure is centred on midnight) to stay consistent with their behaviour.

### Seed size analysis

The average length and width of seeds consumed and dispersed by neotropical frugivores were compiled from studies conducted in French Guiana. The major frugivore species were selected even though we did not observe them in this study. Almost no data existed for toucans in French Guiana. However, the species of toucans from Mata Atlantica are very similar to those from French Guiana because they are either the same species (e.g., *Ramphastos vitellinus*) or species with comparable sizes and body mass (e.g., *Selenidera maculirostris* (160 g) in Mata Atlantica vs *Selenidera piperivora* (150 g) the Guianas). Therefore, we used a dataset for the group Ramphastidae from Mata Atlantica ^83,84^ to compare seed size among frugivores visiting *Virola* trees. Data for *Ramphastos vitellinus*, *Pteroglossus aracari,* and *Selenidera maculirostris* were selected and combined to form the toucans’ group. As normality and homoscedasticity were not verified (tested with Shapiro and Levene test), testing for differences in average seed size consumed by the 9 main arboreal frugivores was done using Kruskall-Wallis tests from the package rstatix^85^ on Rstudio® Version 1.1.463. Then, to identify which paired of frugivores were different, Dunn tests with a Bonferroni correction were done.

All the methods were performed in accordance with relevant guidelines and regulations. All analyses and figures were done and edited on Rstudio® Version 1.1.463.

## Supplementary Information

Supplementary Information.
