# Supplementary material for: Arboreal camera trap reveals the frequent occurrence of a frugivore-carnivore in neotropical nutmeg trees
Source: Sci Rep. 2022 May 7;12:7513. doi: 10.1038/s41598-022-11568-z (PMC9079064; doi:10.1038/s41598-022-11568-z)
Supplement: Supplementary file 1 — Supplementary Information. [file 41598_2022_11568_MOESM1_ESM.pdf]

Arboreal camera-trap reveals the frequent occurrence of a frugivore-carnivore in neotropical nutmeg trees

Marie Seguigne[1,2,*], Opale Coutant[1,3], Benoit Bouton[1,4,5], Lionel Picart[1,4], Éric Guilbert[1] & Pierre-Michel Forget[1]

[1] Mécanismes Adaptatifs et Evolution MECADEV – UMR 7179, CNRS-MNHN, Labex DRIIHM, Département Adaptations du Vivant, Muséum National d'Histoire Naturelle, 1 avenue du petit château, 91800 Brunoy, France

[2] Laboratoire d'Écologie Fonctionnelle et Environnement - UMR 5245, Université Toulouse III Paul Sabatier - Bâtiment 4R1, 118 route de Narbonne, 31062 Toulouse cedex 9, France

[3]Laboratoire Évolution & Diversité Biologique - UMR 5174, Université Toulouse III Paul Sabatier - Bâtiment 4R1, 118, route de Narbonne, 31062 Toulouse cedex 9, France

[4]Hevea Maurepas, ZAC Pariwest, 6 Av. Louis Pasteur, 78310 Maurepas, France

[5]A L'écoute De L'arbre, 3, rue de la fontaine pigeon, 60650 Lachapelle-aux-Pots, France

* Author for correspondence: marie.seguigne@univ-tlse3.fr

Supplementary materials

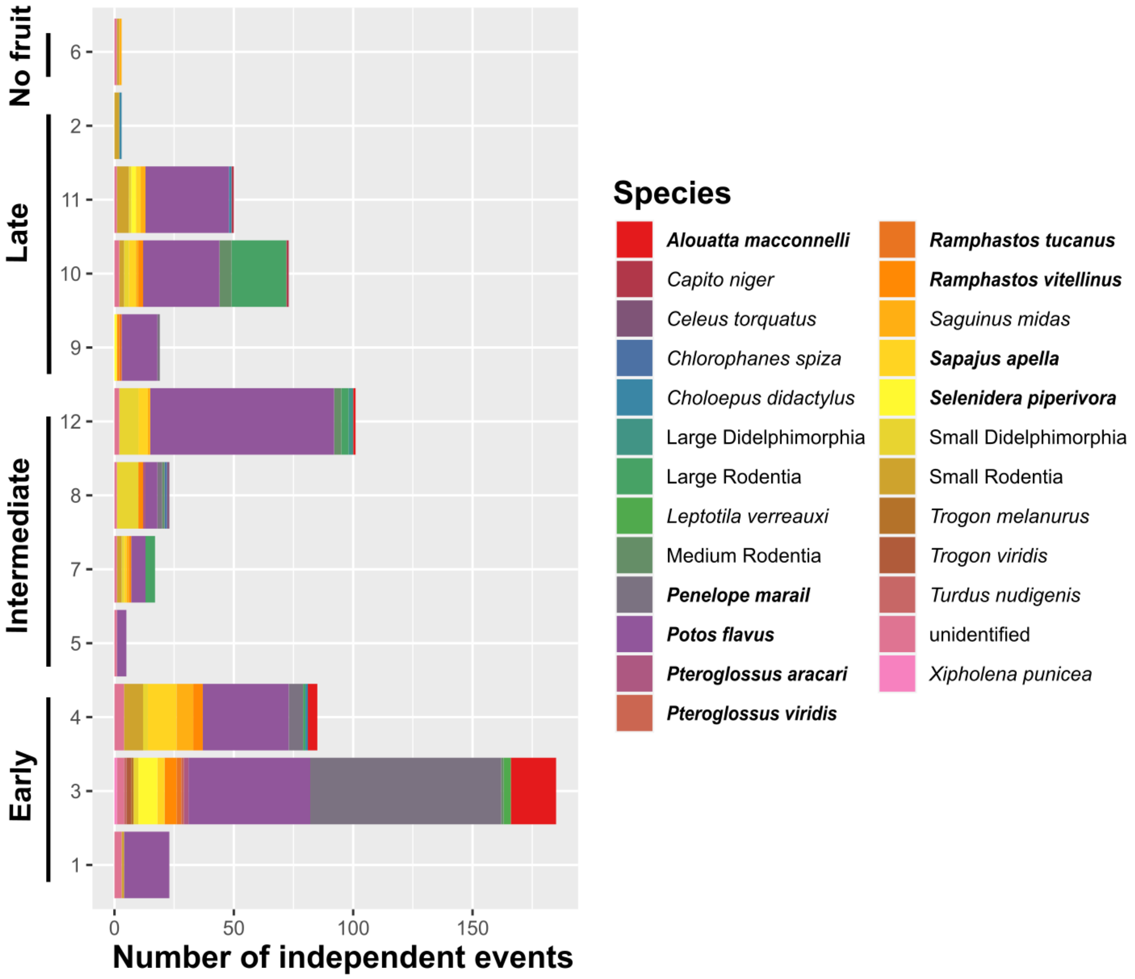

Figure S1. Number of independent events by tree for every species and order identified, recorded as visiting the canopy of nutmeg trees between 12/01/19 and 01/24/20 in National 2 Road in French Guiana. Trees were grouped by their fruiting statues, either "early", "intermediate", "late" or "no fruit". Bold species represent the nine main frugivores observed.

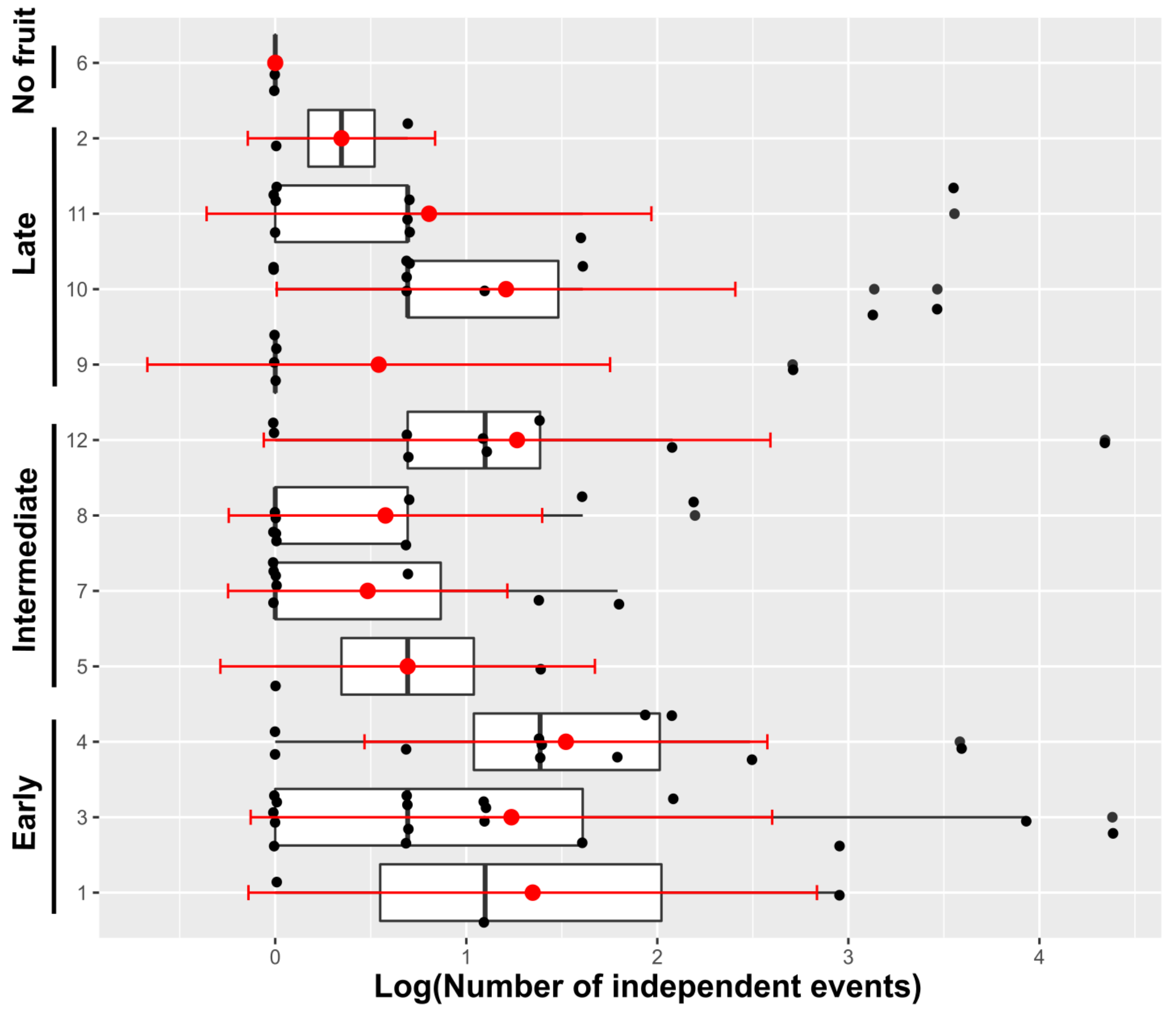

Figure S2. Variability between trees of number of total independent events (log transformed) with error bars and means (dot) in red. Trees were grouped by their fruiting statues, either “early”, “intermediate”, “late” or “no fruit”.

Table S1. Description of independent events rate per tree calculated with the overall number of independent events related to the number of trap days. Details of mean and standard deviation are given, calculated from the total number of events per tree.

Tree	Event rate (%)	Mean	Standard deviation
1	41.82	0.92	3.817940
2	5.46	0.12	0.439697
3	336.36	7.40	18.40516
4	154.546	3.40	7.511103
5	9.09	0.20	0.816497
6	5.45	0.12	0.331663
7	30.91	0.68	1.435270
8	41.82	0.92	2.019076
9	34.55	0.76	2.989983
10	132.73	2.92	7.615335
11	90.91	2.00	6.970175
12	183.64	4.04	15.31470

Figure S3. Photographs of the main 9 frugivores that were observed in fruiting *Virola kwatae* and *V. michelii* trees. (a) *Alouatta macconnelli* ; (b) *Sapajus apella* ; (c) *Potos flavus* ; (d) *Penelope marail* ; (e) *Pteroglossus aracari* ; (f) *Pteroglossus viridis* ; (g) *Ramphastos tucanus* ; (h) *Ramphastos vitellinus* ; (i) *Selenidera piperivora ; (j) P. marail* et *S. piperivora*

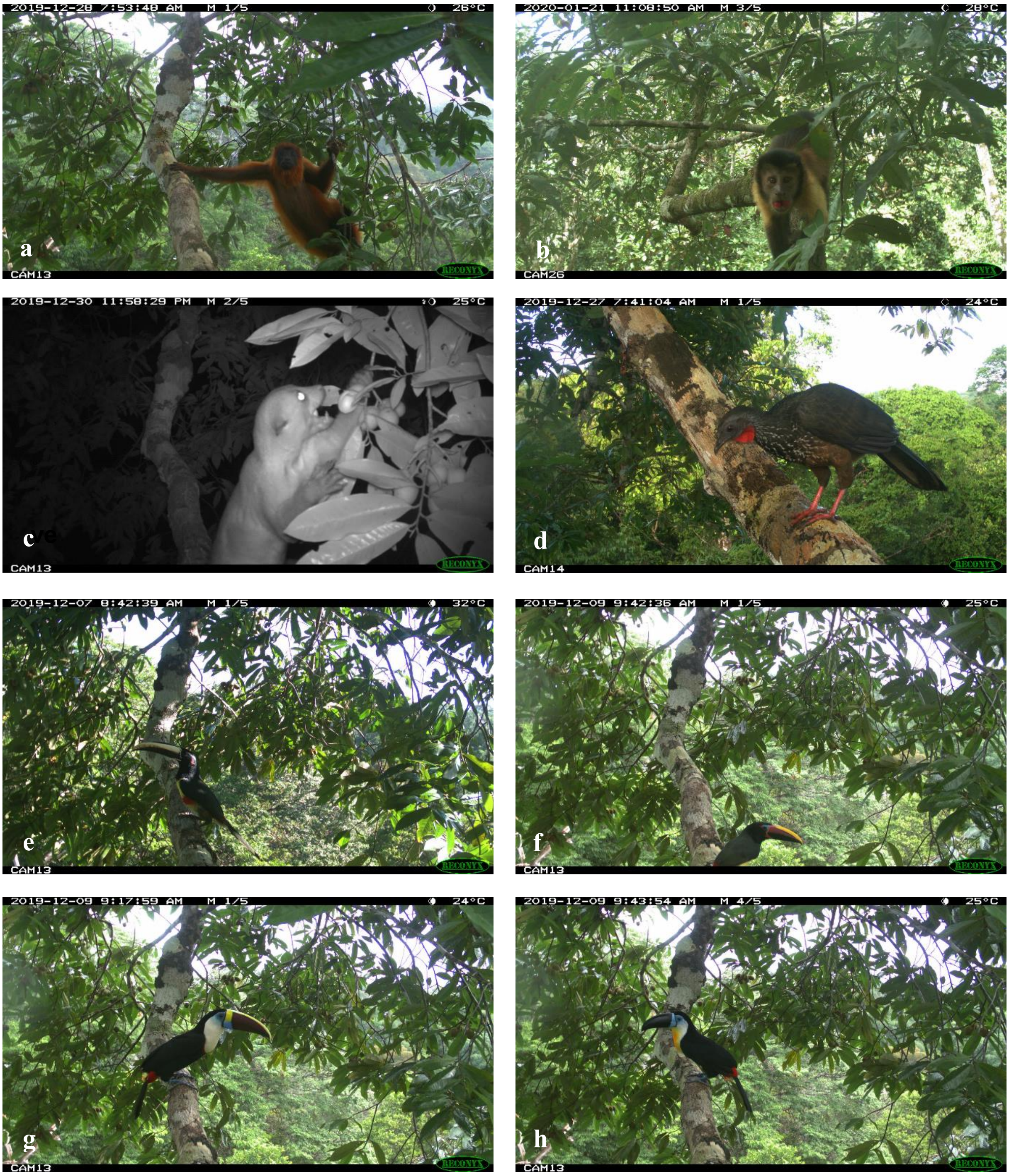

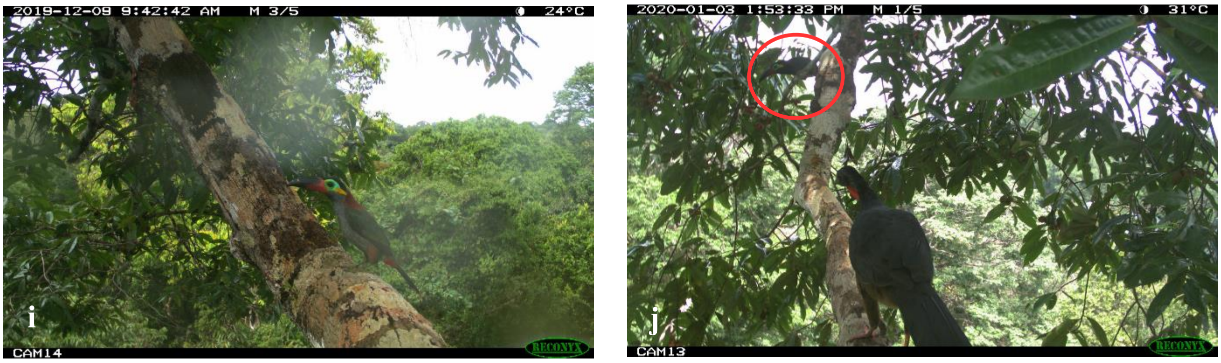

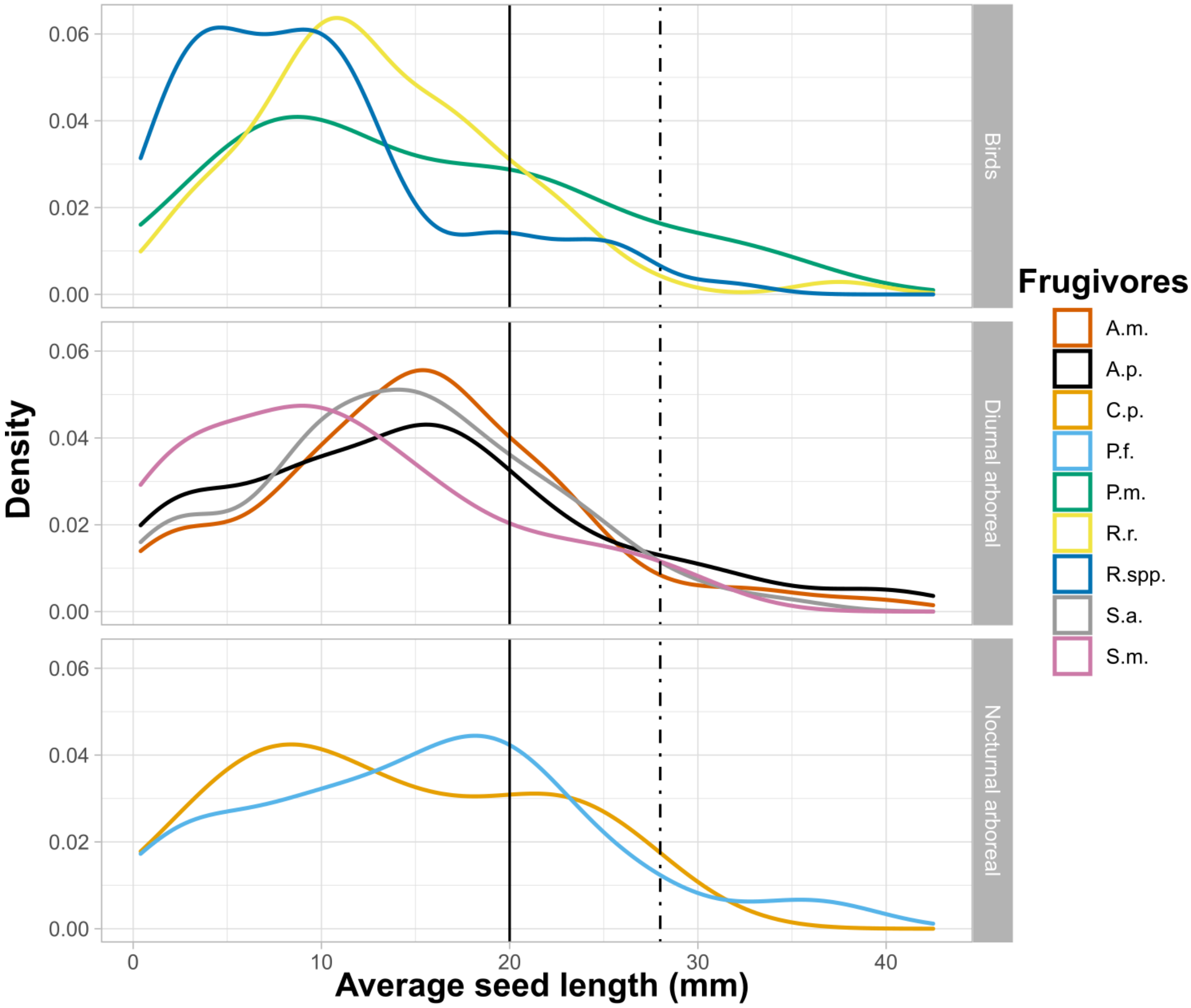

Figure S4. Density plot of average seed length dispersed by the major neotropical arboreal frugivores. Data are obtained from literature based on studies in French Guiana, except for data on Ramphastidae which are compiled from studies in Mata Atlantica. Full black line = average *Virola michelii* seed length. Dashed line = average *Virola kwatae* seed length. *A.m* = *Alouatta macconnelli* ; *A.p.* = *Ateles paniscus* ; *C.p.* = *Caluromys philander* ; *P.f.* = *Potos flavus* ; *P.m.* = *Penelope marail* ; *R.r.* = *Rupicola rupicola* ; *R. spp.* = *Ramphastidae* ; *S.a.* = *Sapajus apella* ; *S.m.* = *Saguinus midas*.

Table S2. Number of events of the main arboreal frugivores observed in each tree sampled. Fruiting stages are classified as such: « A » for the early fruiting stage, « B » for the intermediate fruiting stage, « C » for the late fruiting stage, and « D » for the flowering tree.

		Virola michelii					*Virola kwatae*						
Tree		1	2	3	4	5	6	7	8	9	10	11	12
Fruiting stage		A	C	A	A	B	D	B	B	C	C	C	B
**Mammals**													
Atelidae	*Alouatta macconnelli*	0	0	19	4	0	0	0	1	0	0	0	1
Cebidae	*Sapajus apella*	0	0	3	12	0	0	1	0	0	3	2	4
Procyonidae	*Potos flavus*	19	0	51	36	4	0	6	5	15	32	35	77
**Birds**													
Cracidae	*Penelope marail*	0	0	80	6	0	0	0	2	1	0	0	0
Ramphastidae	*Pteroglossus aracari*	0	0	2	0	0	0	0	1	0	0	0	0
	Pteroglossus viridis	0	0	1	0	0	0	0	0	0	0	0	0
	Ramphastos tucanus	0	0	2	0	0	0	0	0	1	0	0	0
	Ramphastos vitellinus	0	0	5	4	0	0	1	2	1	2	0	0
	Selenidera piperivora	0	0	8	0	0	0	0	0	1	0	2	0

Table S3. Results of Kruskall-Wallis test for average seed width.

	**n**	**Statistic**	**Df**	**Probability**
**Average seed width**	745	35.17697	8	$2.58e^{-05}$

Table S4. P-value from Dunn tests after Bonferroni correction to compare average seed width dispersed by main frugivores. Bold p-values are significant.

	Alouatta macconnelli	***Ateles paniscus***	***Caluromys philander***	***Potos flavus***	***Penelope marail***	***Rupicola rupicola***	***Ramphastidae***	***Sapajus apella***
Ateles paniscus	1.0000							
Caluromys philander	1.0000	1.0000						
Potos flavus	1.0000	1.0000	1.0000					
Penelope marail	1.0000	1.0000	1.0000	1.0000				
Rupicola rupicola	1.0000	1.0000	1.0000	1.0000	1.0000			
Ramphastidae	**0.0007**	**0.0001**	1.0000	**0.0008**	1.0000	0.1371		
Sapajus apella	1.0000	1.0000	1.0000	1.0000	1.0000	1.0000	**0.0251**	
Saguinus midas	0.2005	0.1380	1.0000	0.0989	1.0000	1.0000	1.0000	1.0000

Table S5. Result of Kruskall-Wallis test for average seed length.

	n	Statistic	Df	Probability
**Average seed length**	745	34.23629	8	$3.68e^{-05}$

Table S6. P-value from Dunn test after Bonferroni correction to compare average seed length dispersed by main frugivores. Bold p-values are significant.

	Alouatta macconnelli	*Ateles paniscus*	*Caluromys philander*	*Potos flavus*	*Penelope marail*	*Rupicola rupicola*	*Ramphastidae*	*Sapajus apella*
Ateles paniscus	1.0000							
Caluromys philander	1.0000	1.0000						
Potos flavus	1.0000	1.0000	1.0000					
Penelope marail	1.0000	1.0000	1.0000	1.0000				
Rupicola rupicola	1.0000	1.0000	1.0000	1.0000	1.0000			
Ramphastidae	**0.0001**	**0.0003**	1.0000	**0.0021**	1.0000	0.2489		
Sapajus apella	1.0000	1.0000	1.0000	1.0000	1.0000	1.0000	**0.0007**	
Saguinus midas	0.1436	0.3760	1.0000	0.3622	1.0000	1.0000	1.0000	0.4887

Table S7. Median of average seed width and length (mm) dispersed by the main frugivores and their home-range (N = number of plant species consumed and dispersed)

		Body mass (g)	N	Median width	Median length	Home range (ha)
**Mammals**						
Atelidae	*Alouatta macconnelli*	3600-11000[1]	95	9	15.5	45[2]
	Ateles paniscus	7500-13500[3]	154	9	15	255[4]
						150-230[5]
Cebidae	*Sapajus apella*	1700-4500[6]	122	8	14.5	355[6]
	Saguinus midas	360-660[7]	46	7	10	31.1-42.5[8]
Didelphidae	*Caluromys philander*	300-400[9]	17	8	13.5	3[10]
Procyonidae	*Potos flavus*	2000-3200[11]	54	10	15.5	15.7-39.5[12]
**Birds**						
Cotingidae	*Rupicola rupicola*	140-230[13]	58	8	11.9	40-450[14]
Cracidae	*Penelope marail*	1024-2048[3]	15	10	11.5	ND
Ramphastidae		128-1024[3]	74	5.77	8.5	55-395[15]

References

1. Emmons, L. & Feer, F. *Neotropical Rainforest Mammals: A Field Guide*. vol. 14 (Cambridge University Press, 1998).
2. Julliot, C. Utilisation des ressources alimentaires par le singe hurleur roux, *Alouatta seniculus* (atelidae, primates) en Guyane : Impact de la dissémination des graines sur la regénération forestière. (Tours, 1992).
3. Ratiarison, S. & Forget, P.-M. The role of frugivores in determining seed removal and dispersal in the Neotropical nutmeg. *Trop. Conserv. Sci.* **6**, 690–704 (2013).
4. van Roosmalen, M. G. M. Habitat preferences, diet, feeding strategy and social organization of the black spider monkey [*Ateles paniscus paniscus* Linnaeus 1758] in Surinam. *Acta Amaz.* **15**, 7–238 (1985).
5. Russo, S. E. & Augspurger, C. K. Aggregated seed dispersal by spider monkeys limits recruitment to clumped patterns in *Virola calophylla*. *Ecol. Lett.* **7**, 1058–1067 (2004).
6. Zhang, S.-Y. Activity and ranging patterns in relation to fruit utilization by brown capuchins (*Cebus apella*) in French Guiana. *Int. J. Primatol.* **16**, 489–507 (1995).
7. Richard-Hansen, C., Vie, J.-C., Vidal, N. & Keravec, J. Body measurements on 40 species of mammals from French Guiana. *J. Zool.* **247**, 419–428 (1999).
8. Day, R. T. & Elwood, R. W. Sleeping site selection by the golden-handed tamarin *Saguinus midas midas*: The role of predation risk, proximity to feeding sites, and territorial defence. *Ethology* **105**, 1035–1051 (1999).
9. Atramentowicz, M. Growth of pouch young in the bare-tailed woolly opossum, *Caluromys philander*. *J. Mammal.* **76**, 1213–1219 (1995).
10. Julien-Laferrière, D. Use of space by the woolly opossum *Caluromys philander* (Marsupialia, Didelphidae) in French Guiana. *Can. J. Zool.* **73**, 1280–1289 (1995).
11. Charles-Dominique, P. *et al.* Les mammifères frugivores arboricoles nocturnes d'une forêt guyanaise: Inter-relation plantes-animaux. *Rev. Ecol. Terre Vie* **35**, (1981).

12. Julien-Laferriere, D. Radio-tracking observations on ranging and foraging patterns by kinkajous (*Potos flavus*) in French Guiana. *J. Trop. Ecol.* **9**, 19–32 (1993).

13. Théry, M. & Larpin, D. Seed dispersal and vegetation dynamics at a cock-of-the-rock's lek in the tropical forest of French Guiana. *J. Trop. Ecol.* **9**, 109–116 (1993).

14. Ricardou, A., Lefèvre, S., Luglia, T. & de Pracontal, N. *Guide technique pour la prise en compte du coq-de-roche dans les projets d'aménagements*. https://lifecapdom.org/les-projets/article/le-coq-de-roche-orange (2010).

15. Holbrook, K. M. Home range and movement patterns of toucans: Implications for seed dispersal. *Biotropica* **43**, 357–364 (2011).

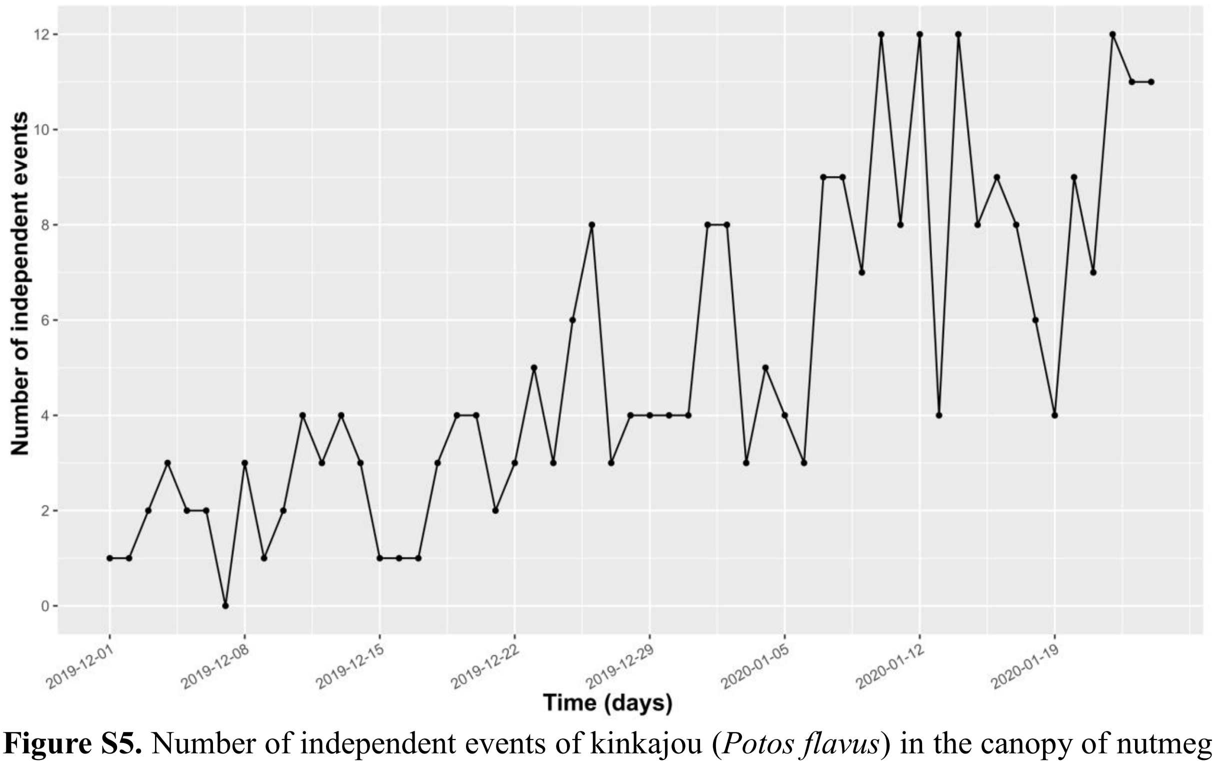

Figure S5. Number of independent events of kinkajou (*Potos flavus*) in the canopy of nutmeg trees by day between the 12/01/19 and the 01/24/20 in National 2 Road in French Guiana.
